# Soft Tissue Interface with Various Kinds of Implant Abutment Materials

**DOI:** 10.3390/jcm10112386

**Published:** 2021-05-28

**Authors:** Akihiro Furuhashi, Yasunori Ayukawa, Ikiru Atsuta, Yunia Dwi Rakhmatia, Kiyoshi Koyano

**Affiliations:** 1 Section of Implant and Rehabilitative Dentistry, Division of Oral Rehabilitation, Faculty of Dental Science, Kyushu University, Fukuoka 812-8582, Japan; ayukawa@dent.kyushu-u.ac.jp (Y.A.); rakhmatia@dent.kyushu-u.ac.jp (Y.D.R.); 2 Division of Advanced Dental Devices and Therapeutics, Faculty of Dental Science, Kyushu University, Fukuoka 812-8582, Japan; atyuta@dent.kyushu-u.ac.jp (I.A.); koyano@dent.kyushu-u.ac.jp (K.K.)

**Keywords:** dental implant, abutment, soft tissue, titanium, zirconia, platinum gold alloy, epithelial cells, fibroblasts, biologic width, supracrestal tissue attachment

## Abstract

Various materials, such as titanium, zirconia and platinum-gold (Pt-Au) alloy, have been utilized for dental implant trans-mucosal parts. However, biological understanding of soft tissue reaction toward these materials is limited. The aim of this study was to compare the response of cell lines and soft tissue to titanium, zirconia and Pt-Au substrata. The surface hydroxyl groups and protein adsorption capacities of the substrata were measured. Next, gingival epithelial-like cells (Sa3) and fibroblastic cells (NIH3T3) were cultured on the materials, and initial cell attachment was measured. Immuno-fluorescent staining of cell adhesion molecules and cytoskeletal proteins was also performed. In the rat model, experimental implants constructed from various materials were inserted into the maxillary tooth extraction socket and the soft tissue was examined histologically and immunohistochemically. No significant differences among the materials were observed regarding the amount of surface hydroxyl groups and protein adsorption capacity. Significantly fewer cells of Sa3 and NIH3T3 adhered to the Pt-Au alloy compared to the other materials. The expression of cell adhesion molecules and a well-developed cytoskeleton was observed, both Sa3 and NIH3T3 on each material. In an animal model, soft tissue with supracrestal tissue attachment was observed around each material. Laminin-5 immuno-reactivity was seen in epithelia on both titanium and zirconia, but only in the bottom of epithelia on Pt-Au alloy. In conclusion, both titanium and zirconia, but not Pt-Au alloy, displayed excellent cell adhesion properties.

## 1. Introduction

Dental implant treatment is a widely accepted prosthodontic procedure. Together with the aging society, demand for long-term stability and effective functional rehabilitation is increasing [1]. Osseointegration has been considered as a fundamental and priority factor related to the success of the implants [2]. Moreover, soft tissue stability around dental implant is one of the important factors for the long term outcome of the dental implant treatment. Repeated chewing cycles may produce abutment loosening and development of a gap between abutment and implant [3]. It is important to gain an understanding of the relation between implant–abutment complex design and load distribution at the bone–implant interface [4]. The different implant–abutment materials and designs have very different characteristics, which can affect their mechanical stability [5].

Various kinds of implant abutment materials such as titanium, cast gold alloy and zirconia are used. Titanium abutment is one of the major parts with a lot of clinical evidence [6,7]. Cast gold abutments can be customized by wax-up and a superstructure can be made in one piece [8]. Because of the current technological advances, such as computer-aided design and computer-aided manufacture (CAD/CAM) technology, and with the escalating price of precious metals, the ceramic zirconia, which elicits high mechanical property for application as an excellent esthetic, is being increasingly used as an implant superstructure material [9].

In the case of natural teeth, adhesion structures like hemidesmosomes, between enamel and the epithelium, play an important role in protection from harmful stimuli from the oral cavity. It is reported that the quantity of hemidesmosomes formed between titanium implants and the epithelium is lower compared to natural teeth [10,11]. This may contribute, in part, to the long-term stability of titanium implants and occurrence of peri-implantitis. In fact, we have shown that improvement of epithelial attachment to dental implants may be a solution to address problems such as peri-implant soft tissue infection and soft tissue recession [12].

It is known that “supracrestal tissue attachment”, which used to be described as “biologic width”, is formed not only around natural teeth, but also dental implants. Titanium, platinum-gold (Pt-Au) alloy and zirconia have been widely used as abutment materials. However, biological evidence is lacking on the interface between the materials and soft tissue. The purpose of the present study was to clarify the difference among the materials toward the surrounding soft tissue.

## 2. Experimental Section

### 2.1. Sample Plates

The tested materials were titanium (commercially pure, grade 4; Niimi, Aichi, Japan), zirconia (Niimi) and Pt-Au alloy (PGA, Ishifuku, Saitama, Japan). The plates were prepared to 1.5 mm in thickness and 5 mm in diameter cylindrical shape. The surface roughness (Ra) was measured (Surftest 501, Mitsutoyo, Kanagawa, Japan) and adjusted to 0.15–0.20 μm.

### 2.2. Quantification of Hydroxyl Groups on Specimens

Quantification of hydroxyl was performed according to the previous report (N = 4) [13]. In short, specimens were soaked for 300 s in a solution of 4 mol/L ammonium chloride and 0.4 mol/L zinc chloride adjusted to 6.9 in pH. Specimens were removed from solution and soaked in distilled water (DW) twice (10 s each) to wash off excess Zn ions that may have attached to the surface without chemical interaction. Specimens were then soaked in nitric acid for 600 s to release chelated Zn ions into the nitric acid solution. Zn ion concentration was determined using an inductively coupled plasma atomic emission spectrometer (Optima 7300 DV, Perkin Elmer, MA, USA) to quantitatively evaluate the number of hydroxyl radicals.

### 2.3. Quantification of Protein Adsorption on Specimens

Specimens were soaked in fetal bovine serum (FBS, Biowest, Nuaillé, France) for 24 h (N = 4). Specimens were then washed with DW to remove excess proteins that may have attached to the surface without chemical interaction. The amount of nitrogen on the surface was measured as an indicator for the amount of adsorbed protein using an X-ray photoelectron spectrometer (XPS, K-Alpha, Thermo Fisher Scientific, Waltham, MA, USA) [14,15].

### 2.4. Initial Cell Attachment Assay

The plates were placed into wells of a 48-well dish and 6 × 10^4^ cell of human oral epithelial-like cells (Sa3, RCB0980, RIKEN, Tsukuba, Japan), in Basal Medium Eagle containing 17% FBS, or fibroblastic cells (NIH3T3, RIKEN), in α-Minimum Essential Medium m (a-MEM, Invitrogen, Carlsbad, CA, USA) containing 10% FBS was dropped on each plate (N = 7). After incubation for 3 h, the relative cell number was measured using the cell count kit (Cell Count Reagent SF, Nacalai Tesque, Kyoto, Japan) [16].

### 2.5. Immunofluorescent Staining

After incubation for 72 h, cells were fixed with 4% formaldehyde (Merck, Darmstadt, Germany) for 10 min, blocked with 1% bovine serum albumin (BSA; Bovine Serum Albumin Fraction V, Roche Diagnostics, Basel, Switzerland) for 30 min at room temperature (RT), and then incubated overnight at 4 °C with a 1:200 dilutions in BSA of goat anti-rat integrin β4 (In-β4) polyclonal antibody (C-20, Santa Cruz Biotechnology, Dallas, TX, USA) for Sa3 and 1:200 dilutions in BSA of goat anti-rat vinculin polyclonal antibody for NIH3T3 (N = 7). After washing with PBS (5 min × 3 times), the cells were labeled for 2 h at RT with a 1:200 dilutions in BSA of FITC-conjugated anti-goat IgG secondary antibody (Invitrogen). Actin filaments were stained for 1 h at RT with a 1:100 dilutions in BSA of tetramethylrhodamine isothiocyanate (TRITC)-conjugated phalloidin (Sigma-Aldrich, St. Louis, MO, USA). The cells were then mounted with anti-fade reagent containing 406-diamidino-2-phenylindole (DAPI; VECTASHIELD, Vector Laboratories, Burlingame, CA, USA) for nuclear staining. The stained cells were observed under a fluorescence microscope (BZ-9000, Keyence, Osaka, Japan).

### 2.6. In Vivo Study and Immunohistochemical Sample Preparation

**Figure 1:**
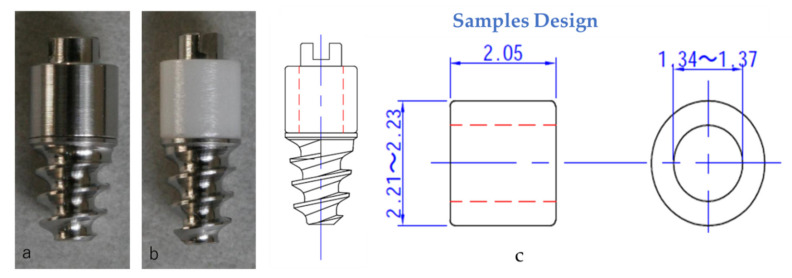
The tested implant abutment materials: titanium (**a**), zirconia (**b**), and samples design (in mm) for each material, including Pt-Au alloy (**c**).

In vivo study was approved by Kyushu University animal experiment ethics committee, approval number: A23-105-0. Titanium experimental implants (Kentec, Tokyo, Japan), 2.2 mm in diameter and 4 mm in length, were fabricated for rat oral implantation. The transmucosal components of the implants were fabricated by pushing cylindrical-shaped 2 mm length titanium, zirconia and Pt-Au alloy into the narrow titanium head (Figure 1). Using 4-week old male Wistar rats, oral implantation was carried out as described previously (N = 6, for each test group) [10,11]. After healing for 4 weeks, rats were euthanized, fixed and maxillae containing implants and surrounding soft tissue were harvested. After demineralization using EDTA solution, implants and hard tissue were removed. Soft tissue samples were snap frozen after being embedded in 20% sucrose overnight at 4 °C, immersed in O.C.T. compound (Sakura Finetek, Tokyo, Japan) for 2 h at 4 °C, cut buccopalatally into 10 μm thick cryosections. For histology, sections were stained with hematoxylin to observe the histological structures. For immunohistochemical staining, sections were blocked for 30 min with 10% normal goat serum and incubated overnight with polyclonal rabbit Ln-5 IgG (Clone 2778; provided by Dr. Vito Quaranta, The Scripps Research Institute, La Jolla, CA, USA; 1:100) at 4 °C, followed by biotinylated goat anti-rabbit IgG (1:200) for 45 min, and visualized with 3′-3′-Diaminobenzidine to observe the presence of laminin-5. After counterstaining with hematoxylin, samples were observed under microscope (BZ-9000).

### 2.7. Statistical Analysis

This test procedure was performed for quantification hydroxyl groups, quantification of protein adsorption, and initial cells attachment. Data are expressed as the mean ± standard deviation (SD). One-way analysis of variance (ANOVA) with Tukey’s method (for multiple comparison) was performed. Values of *p* < 0.05 were considered statistically significant.

## 3. Results

### 3.1. Presence of Hydroxyl Groups on Specimens

**Figure 2:**
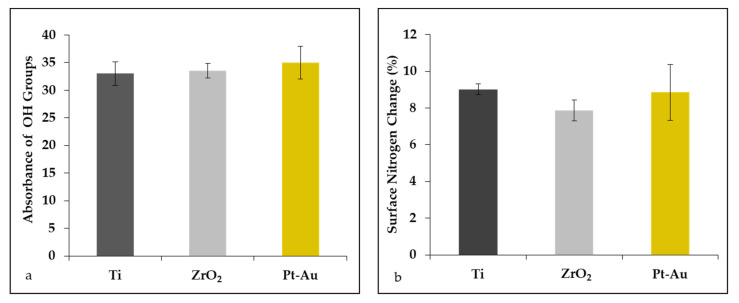
Absorbance of hydroxyl group (**a**) and surface nitrogen change (**b**) on the surface in each group. (N = 4; ANOVA, Tukey).

The presence of hydroxyl groups on the surface was detected on each group (Figure 2a). There was no significant difference of the amount of the hydroxyl group among the group.

### 3.2. Protein Adsorption on Specimens

The presence of nitrogen on the surface was detected on each group (Figure 2b). There was no significant difference of the amount of the nitrogen among the group. Adsorption was still permitted after soaking in DW.

### 3.3. Initial Cell Attachment

**Figure 3:**
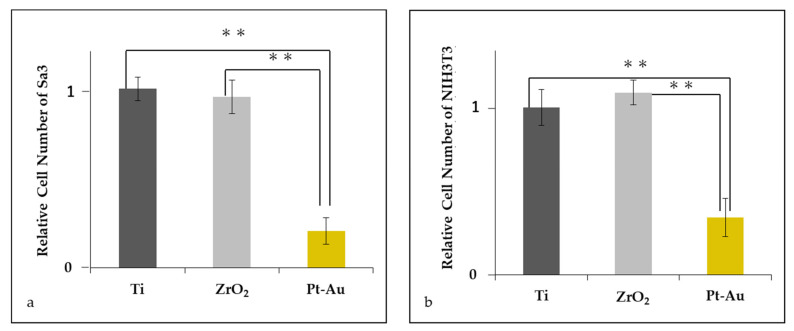
Relative cell number of Sa3 (**a**) and NIH3T3 (**b**) on the surface in each group (Average value of Ti = 1). (N = 7; ANOVA, Tukey, ** *p* < 0.01).

Significantly fewer cells adhered to Pt-Au alloy compared to the other materials in both Sa3 (Figure 3a) and NIH3T3 (Figure 3b).

### 3.4. Immuno-Fluorescent Findings of the Cells

**Figure 4:**
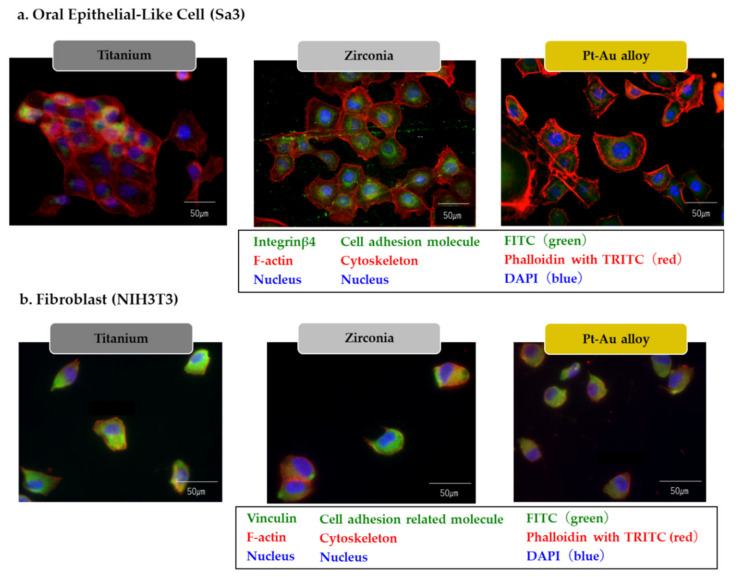
Immuno-fluorescent finding of Sa3 (**a**) and NIH3T3 (**b**) on the surface in each group (Bar = 50 μm).

Well-developed cytoskeleton of actin filament was observed in both Sa3 and NIH3T3, meanwhile expression of adhesion protein of In-β4 was observed for Sa3 (Figure 4a) and vinculin for NIH3T3 (Figure 4b). No clear difference was seen among the groups.

### 3.5. In Vivo Assessment

**Figure 5:**
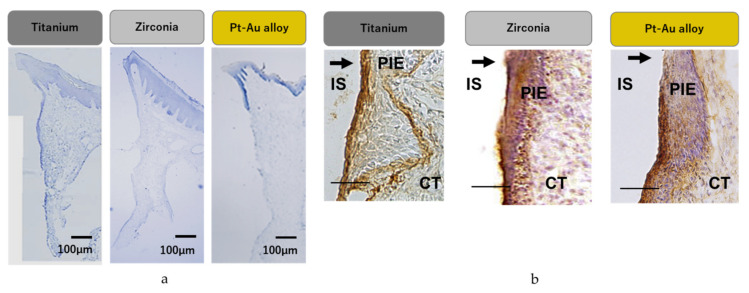
Histology of supracrestal tissue attachment (**a**), (Bar = 100 μm) and immunoreactivity to laminin-5 between the implant–epithelium interface (**b**), (Bar = 50 μm). Expression of laminin-5 (brown staining) at the interface of each material. Strong expression was seen at the titanium and zirconia to epithelium (arrow). Expression of laminin-5 was not evident at the coronal part of the epithelium toward Pt-Au alloy (arrow). IS: implant space (removed), PIE: peri-implant epithelium, CT: connective tissue.

Implant movement and macroscopic inflammation were negligible in the rat implant model 4 weeks following implantation. Each material and soft tissue was observed to be in close contact. Histological staining showed absence of inflammatory cells at the interface between the materials and soft tissue and intercellular space was not increased. The specimen showed similar histological findings. Soft tissue showed normal supracrestal tissue attachment consisting of peri-implant sulcus, epithelial tissue and connective tissue from the coronal side to the crestal bone (Figure 5a).

### 3.6. Immuno-Histochemical Staining

Immunoreactivity to laminin-5, which is typically observed in cell adhesion structure internal basal laminae, was observed at the interface between titanium, zirconia and the surrounding epithelium, whereas it was observed only in the bottom of the epithelium and Pt-Au alloy (Figure 5b).

## 4. Discussion

It has been reported that the surface texture of the substrata affects the response of the surrounding soft and hard tissue [16,17,18]. In order to avoid the effect from the surface texture of the materials, surface roughness was adjusted to the clinically used abutments [19]. Therefore, if specimens with a rougher or smoother surface are used, the result may change. In general, it is known that a rougher surface has a negative effect on the soft tissue [16,18].

Hydroxyl groups are known to correlate to the surface wettability and to the protein adsorption capability of materials [20]. In the present study, hydroxyl groups were observed on each material, and no significant difference of the amount was seen. This result was consistent with the protein adsorption assay. For cell attachment to the extracellular matrix (ECM), prior protein adsorption is a prerequisite [21]. Cells have surface trans-membrane adhesion molecules, including integrins, which facilitate attachment to the ECM surface. Most human cells proliferate, migrate and differentiate after adherence to the ECM via their adhesion proteins. We therefore examined the protein adsorption property of each surface. FBS is a multiple protein complex and was used to simulate the clinical situation, which was direct contact with the blood in the present study. Because all proteins have a nitrogen component, we evaluated the amount of adsorbed protein by measuring nitrogen. The presence of protein on the materials after soaking in serum may enable the cells to attach to the metal surface via the protein. The interface between serum and metal surface is mainly defined by proteins which adsorb immediately after implantation from biological fluids and blood, forming a layer on the metal surface. Aspect ratio of surface features, hydroxylation and OH groups increased the wettability and could change how proteins accommodate on the metal surface [22].

It was investigated in vitro whether the oral soft tissue cells, epithelial cells and fibroblasts can attach to the materials. We showed the localization of In-β4, an epithelial cell-specific adhesion molecule, and vinculin, an adhesion-related molecule in fibroblasts, which indicate the cellular attachment to the materials. In addition, well developed actin filament, a fundamental cytoskeleton component involving cell locomotion and proliferation [23], was observed. These results indicate that the soft tissue cells can attach and express biological functions on the materials.

However, both epithelial cells and fibroblasts showed significantly lower initial attachment to the Pt-Au alloy compared to titanium and zirconia. It has reported that copper ion released from metal shows cytotoxicity [24]. The Pt-Au alloy we used in this study, which is clinically used in daily practice, contains 20% of copper in the alloy. This might be one of the reasons why soft tissue cells showed a significantly lower attachment to the Pt-Au alloy.

Based on these results, we studied the interface between the materials and epithelium using a rat oral implant model. The biologic width is defined as the dimension of the soft tissue that is attached to the portion of the tooth coronal to the crest of the alveolar bone [25]. Recently, the term biologic width is replaced by a supracrestal tissue attachment consisting of junctional epithelium and supracrestal connective tissue [26]. From the histological findings, a normal supracrestal tissue attachment, which used to be described as biologic width, was observed. This indicated that the biological attachment is formed between the soft tissue and the materials. This is consistent with the meta-analysis in which titanium and zirconia abutment materials contribute to high survival rate of dental implant [27]. We have reported epithelial attachment to titanium and zirconia [28,29] but little is known about the Pt-Au alloy. A previous study found that the tissues around the gold alloy abutment showed a more negative reaction than titanium and zirconia abutments. The Pt-Au alloy was demonstrated to have less attachment resistance and a higher inflammatory response compared with titanium and zirconia abutments [30].

At the interface between enamel and epithelium, the hemidesmosome is formed and both In-β4 and laminin-5 are the major components of this [31]. Our immune-histochemical observation revealed an expression of laminin-5 at the interface, indicating active adhesion of epithelial tissue to the materials. However, the expression was observed only in the bottom part of the epithelium, towards the Pt-Au alloy, which indicates weaker adhesion to the epithelium to the Pt-Au alloy compared to the titanium and zirconia. This may lead to the result of soft/hard tissue apical shift, which has been reported previously [30,32].

## 5. Conclusions

Significantly lower initial cell attachment to Pt-Au alloy was shown compared to titanium and zirconia. However, once attached, the cells expressed adhesion molecules and cytoskeleton on all materials. A supracrestal tissue attachment was histologically observed in all materials. From the immune-histochemical result, expression of laminin-5 in the epithelium was lower in the Pt-Au alloy compared to titanium and zirconia, which may indicate weaker epithelial attachment to the Pt-Au alloy. This would indicate the potential risk for the long term stability of the healthy soft tissue around the Pt-Au alloy following the outcome of the dental implant treatment. Further study with a clinical evaluation should be conducted.

## Data Availability

The data presented in this study are available on request from the corresponding author.
